# DNEA: an R package for fast and versatile data-driven network analysis of metabolomics data

**DOI:** 10.1186/s12859-024-05994-1

**Published:** 2024-12-18

**Authors:** Christopher Patsalis, Gayatri Iyer, Marci Brandenburg, Alla Karnovsky, George Michailidis

**Affiliations:** 1 Department of Computational Medicine and Bioinformatics, University of Michigan, Ann Arbor, MI 48109 USA; 2 Taubman Health Sciences Library, University of Michigan, Ann Arbor, MI 48109 USA; 3 Department of Internal Medicine, Hematology/Oncology Division, University of Michigan, Ann Arbor, MI 48109 USA; 4 Department of Statistics, University of Florida, Gainesville, FL 32611 USA

**Keywords:** Network analysis, Pathway analysis, Partial correlation, Metabolomics, Enrichment analysis, Network visualization

## Abstract

**Background:**

Metabolomics is a high-throughput technology that measures small molecule metabolites in cells, tissues or biofluids. Analysis of metabolomics data is a multi-step process that involves data processing, quality control and normalization, followed by statistical and bioinformatics analysis. The latter step often involves pathway analysis to aid biological interpretation of the data. This approach is limited to endogenous metabolites that can be readily mapped to metabolic pathways. An alternative to pathway analysis that can be used for any classes of metabolites, including unknown compounds that are ubiquitous in untargeted metabolomics data, involves defining metabolite-metabolite interactions using experimental data. Our group has developed several network-based methods that use partial correlations of experimentally determined metabolite measurements. These were implemented in CorrelationCalculator and Filigree, two software tools for the analysis of metabolomics data we developed previously. The latter tool implements the Differential Network Enrichment Analysis (DNEA) algorithm. This analysis is useful for building differential networks from metabolomics data containing two experimental groups and identifying differentially enriched metabolic modules. While Filigree is a user-friendly tool, it has certain limitations when used for the analysis of large-scale metabolomics datasets.

**Results:**

We developed the DNEA R package for the data-driven network analysis of metabolomics data. We present the DNEA workflow and functionality, algorithm enhancements implemented with respect to the package’s predecessor, Filigree, and discuss best practices for analyses. We tested the performance of the DNEA R package and illustrated its features using publicly available metabolomics data from the environmental determinants of diabetes in the young. To our knowledge, this package is the only publicly available tool designed for the construction of biological networks and subsequent enrichment testing for datasets containing exogenous, secondary, and unknown compounds. This greatly expands the scope of traditional enrichment analysis tools that can be used to analyze a relatively small set of well-annotated metabolites.

**Conclusions:**

The DNEA R package is a more flexible and powerful implementation of our previously published software tool, Filigree. The modular structure of the package, along with the parallel processing framework built into the most computationally extensive steps of the algorithm, make it a powerful tool for the analysis of large and complex metabolomics datasets.

**Supplementary Information:**

The online version contains supplementary material available at 10.1186/s12859-024-05994-1.

## Background

Metabolomics is a high-throughput technology for measuring small molecule metabolites in cells, tissues or biofluids. It is widely used in biomarker discovery and mechanistic studies aimed at understanding physiological and pathological processes as well as assessing response to environmental stimuli. Depending on whether a targeted or untargeted approach is used, hundreds to thousands of metabolites can be measured [1]. Analysis of such data is a multi-step process that involves data processing, quality control and normalization, followed by statistical and bioinformatics analysis. The latter step often involves pathway analysis to aid biological interpretation of the data [2]. Pathway analysis works well for endogenous metabolites that can be mapped to metabolic pathways (e.g. primary metabolites). However, the coverage of lipid and secondary metabolism by pathway databases is scarce, which limits its usefulness for the analysis of these metabolites. Further, unknown metabolites detected by untargeted metabolomics studies and exogenous metabolites that are routinely measured by metabolomics assays cannot be mapped to metabolic pathways.

An alternative approach to pathway analysis that can overcome the aforementioned limitations involves defining metabolite-metabolite interactions using experimental data [3]. Data-driven approaches have been used in a number of biomedical applications [4–12]. The goal of these approaches is to use some measure of statistical association (e.g., Pearson correlations, partial correlations, mutual information, etc.) to build a metabolite network. Pearson correlation networks are easy to obtain, but they result in dense networks that capture both direct and indirect associations between metabolites. In contrast, partial correlation networks capture associations between a pair of metabolites, while also controlling for the influence of all other metabolites in a dataset. These techniques allow for more meaningful networks to be constructed, which contain potential direct metabolic reaction partners [13]. However, to obtain a partial correlation network when the number of metabolites measured in the dataset exceeds the number of the biological samples, *regularization* techniques need to be employed to avoid spurious connections [14].

Our group has developed several tools for constructing data-driven partial correlation networks from metabolomics data [9, 15, 16]. One of these tools, CorrelationCalculator, can estimate a partial correlation network (PCN) and provide confidence intervals for the discovered edges from high-dimensional metabolomics and lipidomics data when the number of samples (n) meets or exceeds the number of metabolites (m) [15]. Many metabolomics studies aim to compare metabolite levels in *two or more* experimental groups. The CorrelationCalculator can be used to estimate networks for each group separately, however, the number of samples per group often becomes a limiting factor. To address this issue, our group developed the Differential Network Enrichment Analysis (DNEA) algorithm [9]. DNEA performs joint network estimation across two experimental groups, taking advantage of all available samples. Regularization, through both a penalty parameter [17] and data resampling techniques [18], are utilized to avoid the inclusion of false positive edges and enable the analysis of datasets where the number of features exceeds the number of samples. The network estimation step is followed by consensus clustering of the resulting network to identify highly interconnected subnetworks. The final step of the DNEA algorithm tests the resulting subnetworks, or metabolic modules, for enrichment using the NetGSA algorithm [19, 20]. This workflow is implemented in a Java-based tool, named Filigree [16]. The convenient user-interface and detailed documentation makes the program suitable for users at all levels [21]. However, certain components of the DNEA algorithm require considerable computing resources. As metabolomics datasets become larger both in terms of the number of metabolites and the number of samples being analyzed within a given experiment, the computing power available to a user on a personal computer becomes a limiting factor in analyses. To address this issue, we developed an R package that implements the DNEA algorithm while maintaining the simplicity and usability of Filigree’s user-interface. Further, the DNEA R package has many additional features, including parallel-processing capability, an improved algorithm to aggregate highly correlated metabolites into singular features, and a modularized workflow that allows the user to customize the analysis to their preferences. The package can be utilized on cloud-computing services or other high-performance computing systems running the R statistical programming language. We used publicly available metabolomics data from the Environmental Determinants of Diabetes in the Young (TEDDY) study to test the DNEA R package and to illustrate its features [22].

### Design and implementation

A detailed description of the DNEA algorithm has been published previously [9]. Here we describe the algorithm’s implementation in the DNEA R package and focus on specific improvements. The package workflow is built around a custom object, *DNEAobj*. The object-oriented nature of this implementation maintains an intuitive design for all users, similar to the previous implementation [16]. The workflow can be divided into four main steps: (1) Data pre-processing and optional feature aggregation, (2) Model tuning via hyperparameter tuning and stability selection, (3) network estimation and consensus clustering, and (4) enrichment analysis via NetGSA and network visualization.

Step 1. Data pre-processing and feature aggregation

#### Data pre-processing

*INPUT—*The workflow is initiated by the createDNEAobject() function. The required input for this function is an *m x n* table that includes measurements for each metabolite (*m*) as rows and the samples (*n*) as columns. DNEA can analyze data from two experimental groups. A list of experimental group labels (i.e., case vs. control) that corresponds to the samples in the dataset must be provided.

When a custo*m DNEAobj* object is created, the input data are log-transformed and differential analysis is performed using a Student’s t-test. Recognizing that alternative normalization methods can be employed for metabolomics data (e.g. square or cubic root transformation etc.), we provide the user with an option to input normalized data following the initiation of the *DNEAobj* object.

The networks are constructed using the joint estimation method (JEM) as described in Ma et al. [9], where a regularized model is fitted for each experimental group separately (while the tuning parameter incorporates information from both groups). An underlying assumption of the JEM is that data are centered and standardized, such that the measurements for each metabolite have mean 0 and unit variance. The data are standardized for each experimental group separately. As a result, the mean expression level for each group is centered at zero. While the user has an option to input standardized data, in order to perform accurate differential analysis, un-scaled data must be provided.

Next, a diagnostic test is conducted by computing the Pearson correlation matrix using the normalized data and performing an eigen decomposition. If the smallest eigenvalues are negative or very close to zero (or equivalently the condition matrix of each correlation matrix is extremely large), it is recommended that the user considers performing feature aggregation *(STEP 1.2)* before continuing the analysis.

*OUTPUT* The output of the createDNEAobject() function is a *DNEAobj* object that contains the normalized data and results of the Differential Expression analysis in the node_list slot of the object. This object is then passed to functions downstream that perform the subsequent steps of the workflow and save their results to the object.

#### Feature aggregation

*INPUT—*Feature aggregation is an optional step performed by passing the *DNEAobj* object to the aggregateFeatures() function. Three feature aggregation methods are available: a correlation-based, a knowledge-based, and a hybrid method. These three methods have been described previously [16]. Briefly, the correlation-based method uses Pearson correlations to aggregate highly correlated metabolites into singular features. The knowledge-based method uses user-defined groups of metabolites/lipids (e.g. chemical classes, metabolic pathways etc.), while the hybrid approach aggregates highly correlated features that belong to the same group. The implementation of the correlation-based and the hybrid methods have been modified to allow the user to apply a desired threshold on the Pearson correlations.

Feature aggregation may be beneficial to the analysis for several reasons, namely simplifying network interpretation and improving performance of the algorithm. The regularization steps employed in DNEA can automatically correct instability to some extent, however, the user can address this issue by aggregating highly correlated metabolites—particularly if the data set contains many features of the same class of compounds (i.e. fatty acids, carnitines, etc.). Network density scales with the size of the dataset and aggregating chemically similar metabolites that are highly correlated may result in more interpretable networks. Additionally, the processing time for a given dataset increases dramatically with an increased number of metabolites. If network resolution at the level of individual metabolites within a given class is not important, aggregating metabolites into highly related features should be considered. In summary, feature aggregation gives the user more control over network construction and simplifies the network interpretation so that meaningful biological associations can be identified more clearly.

*OUTPUT*: The output of this step is a *collapsed_DNEAobj* object wherein features that are highly correlated and/or of the same chemical class are grouped into singular features. The intensity of the aggregate feature is represented as a mean intensity of component features across all samples in each experimental group, respectively. The output data structure inherits the characteristics of the *DNEAobj* object with the addition of a new feature_membership slot that contains information about the aggregated groups of features.

Step 2. Model tuning

Model tuning consists of two steps. First, the Bayesian-Information Criterion (BIC) score is used to optimize the tuning parameter of the regularization procedure utilized in network estimation (*STEP 2.1*) [23]. Next, Stability Selection is performed by randomly sampling the input groups (with optional subsampling of the larger group) and fitting a regularized model to estimate the network (*STEP 2.2*) for a user-specified number of replicates. The results of every random replicate are then summed to calculate the probability that an edge is present between metabolites in any given randomly sampled subset of data (referred to as the selection probability), which are used to obtain the final network.

#### Hyperparameter tuning via BIC

*INPUT—*Hyperparameter tuning is performed by passing the output from *STEP 1* to the BICtune() function.

This step involves optimizing the regularization parameter, lambda (λ). λ can take on a value between 0 and 1. As the value of λ increases model regularization increases, adding sparsity to the network (Supplementary Fig. 1a). Two methods are available to optimize the selection of the model, both of which calculate BIC scores using the methodology developed previously [23] and adopted in Filigree [16]. The λ with the minimum BIC score is the optimal parameter (Supplementary Fig. 1b). The first approach estimates a range for the optimal λ parameter, and then an exhaustive search of values in this range is performed. The second option takes advantage of the mathematical properties of the regularization parameter for datasets with many samples and many features by optimizing the c constant for the asymptotically valid λ, following Eq. 1.

1 $$\lambda = c*\sqrt{\frac{log\left(num. features\right)}{num.samples}}$$

c = values ranging 0.01 to 0.3, incremented by 0.02.

**Fig. 1:**
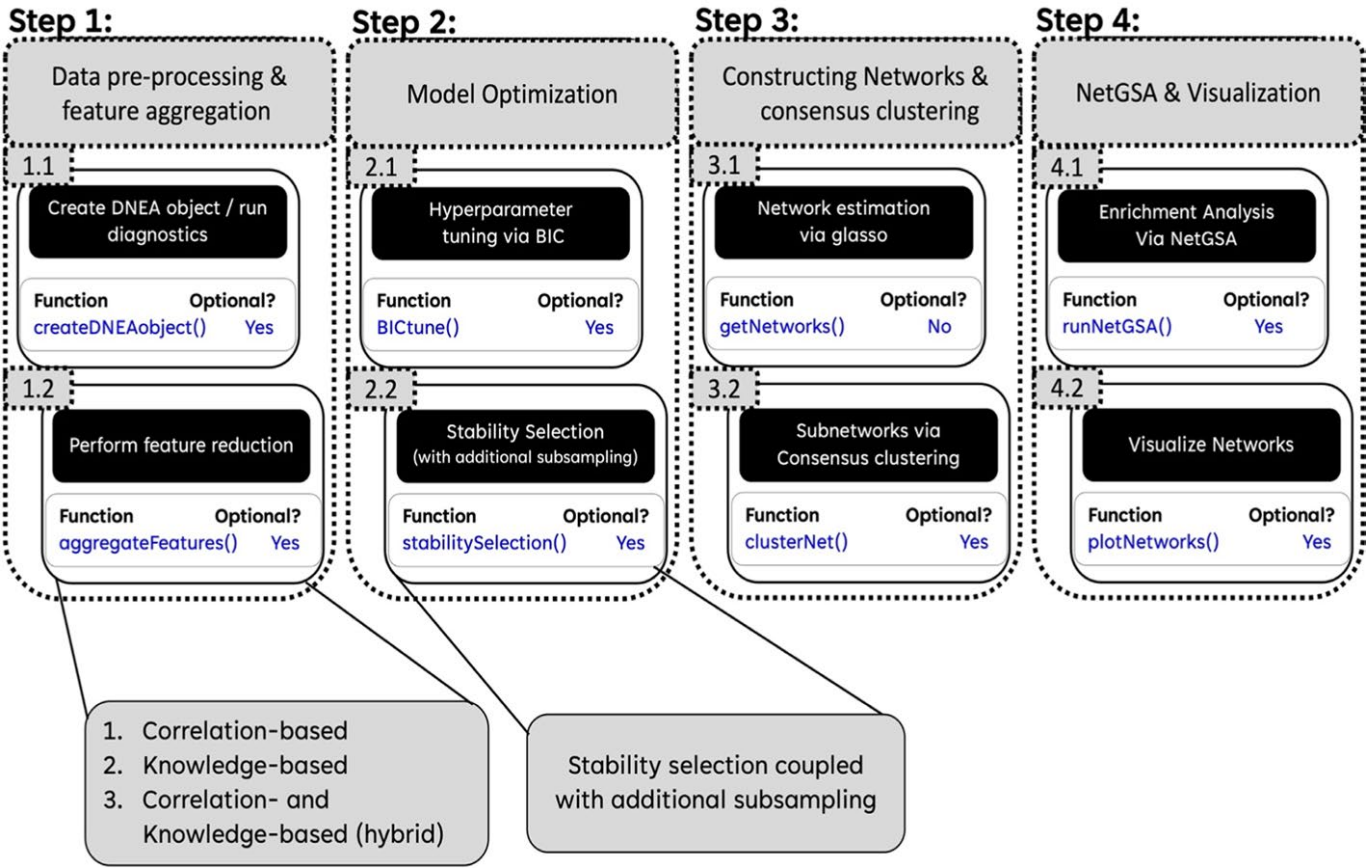
DNEA R package workflow. *(STEP 1.1)* The first step of DNEA takes an *m x n* matrix of peak intensities or concentrations and performs diagnostic testing of the dataset followed differential expression analysis for each metabolite**.** *(STEP 1.2)* Feature reduction may be required if the diagnostic tests determine that the dataset exhibits strong multicollinearity. The user may also choose to perform this step if their dataset contains more features than samples. *(STEP 2.1)* The Bayesian-Information Criterion (BIC) score is used to optimize the tuning parameter λ of the graphical lasso (GLasso) model. *(STEP 2.2)* Stability selection randomly samples the input groups to calculate selection probabilities for each metabolite. *(STEP 3.1)* Network estimation is performed via GLasso utilizing the selection probabilities from step 2. Given sufficient sample size, λ can be tuned for each experimental condition independently or estimated via $\uplambda =\sqrt{\frac{\text{log}\left(m\right)}{n}}$. *(STEP 3.2)* Metabolic modules within the larger networks are identified via a built-in consensus clustering algorithm. *(STEP 4.1)* Pathway enrichment analysis is performed using the NetGSA algorithm to determine differential enrichment in the metabolic modules across the two conditions. *(STEP 4.2)* The constructed networks can then be visualized for interpretation

By utilizing this property of λ, we can reduce the number of computations and time necessary for optimization. Users also have the option to input a list of lambda values to test or optimize the parameter using another method, such as cross-validation, and specify the lambda value to use during modeling.

*OUTPUT—*The function outputs the *DNEAobj* object after populating the hyperparameter slot with the results of parameter optimization, including the optimized λ value.

#### Stability selection

*INPUT*: Stability Selection is performed by passing the *DNEAobj* object created in the previous step to the stabilitySelection() function.

The samples for each group are randomly sampled and a regularized model is fitted for each replicate (we recommend using 1000 replicates) to calculate the selection probability for every potential metabolite-metabolite interaction. When constructing the networks in *STEP 3.1*, the specified λ regularization parameter is weighted for each connection, following Eq. 2.

2 $$\rho =\lambda *(\frac{1}{1e-4* estimated\;selection\;probability })$$

$\lambda$ = regularization parameter, ρ = weighted regularization parameter.

The regularization parameter, rho (ρ), is then used for the final model. This method helps overcome a common problem seen in Omics datasets, where the number of features (in our case metabolites) far exceeds the number of samples, by providing more stringent feature selection than L1 regularization alone. Stability selection improves performance of the algorithm by identifying and removing unstable edges from the final network (Supplementary Fig. 1c). Jointly estimating the selection probabilities in this manner increases the statistical power to accurately quantify common edges amongst the two experimental conditions by utilizing all of the available data.

It is common in biological and clinical data to have experimental groups of uneven size. If the group size is considerably unbalanced, it may result in the inclusion of unstable edges in the estimated networks. To address this issue, we implemented an optional subsampling procedure, as described in Iyer et al. (2).

*OUTPUT*: This step populates the stable_networks slot of the *DNEAobj* object with the results of stability selection as well as the calculated selection probabilities.

Step 3. Network construction and consensus clustering

Network estimation is performed via a regularized procedure (*STEP 3.1*) utilizing the optimized tuning parameters and selection probabilities from *STEP 2*. The consensus clustering algorithm can then be used to identify metabolic modules within the larger networks, as described in Ma et al. [9] (*STEP 3.2*).

#### Network construction

*INPUT—*The data-driven networks are constructed by passing the *DNEAobj* object obtained from the previous steps to the getNetworks() function.

In most cases, BIC tuning and stability selection should be performed, however, for large datasets where the number of samples far exceeds the number of metabolites, particularly for curated datasets, the modularity of the DNEA R package now enables the user to bypass STEP 2 to construct a simple partial correlation network instead. At this step, the independent networks are constructed for each experimental group and the λ parameter is optimized for each group. Depending on the size of the dataset, there are two options for optimizing the λ parameter. If the dataset is sufficiently large (i.e. contains more than 500 samples per group) the BIC tuning protocol can be used to optimize the λ parameter for each experimental condition. This method improves the sensitivity of the analysis for large datasets, however, also tends to increase the density of resulting networks. To address this, the filterNetworks() function is available to filter networks based on edge strength (percentile or partial correlation threshold) and highlight the most biologically relevant connections. For smaller datasets, approximating λ provides stronger regularization that simplifies networks and reduces false positive edges. If the dataset has between 50 to 500 samples per group, the λ parameter should be approximated following Eq. 3:

3 $$\lambda = \sqrt{\frac{log\left(\# features\right)}{\# samples}}$$

$$\lambda$$ = regularization parameter.

*OUTPUT –* Once the networks are constructed, an edge list will be generated and stored in the edge_list slot of the *DNEAobj* object. The resulting table will contain information about the network edges, including partial correlation values for each experimental condition.

#### Consensus clustering

The resulting networks (even those derived from moderately-sized datasets) can be quite dense, complicating the task of visually identifying relevant interactions. To address this issue, it is beneficial to cluster the metabolites within the estimated networks to create metabolic modules that can then be tested for enrichment across experimental conditions, as described below.

*INPUT—*This step is initiated by passing the *DNEAobj* object from *STEP 3.1* to clusterNet().

We employ a consensus clustering algorithm described in Ma et al. [9]. clusterNet() runs seven widely used clustering algorithms from the igraph R package: cluster_edge_betweeness, cluster_fast_greedy, cluster_infomap, cluster_label_prop, cluster_louvain, cluster_walktrap, and cluster_leading_eigen. Consensus clustering is performed to identify subnetworks of highly interconnected metabolites with direct interaction relationships. By default, the agreement threshold, or the percentage of clustering algorithms that must agree on the membership of a given subnetwork, is set to a minimum of 50% (i.e. 4 of the 7 clustering algorithms must agree). The user may prefer a more stringent cutoff and can increase the specificity by providing any value between 0.5–1 as the tau (τ) parameter. Increasing τ requires stronger agreement among clustering methods for a node to be included in a metabolic module. Generally, the number of metabolic modules increases with τ, while the number of nodes per module decreases (Supplementary Fig. 2).

**Fig. 2:**
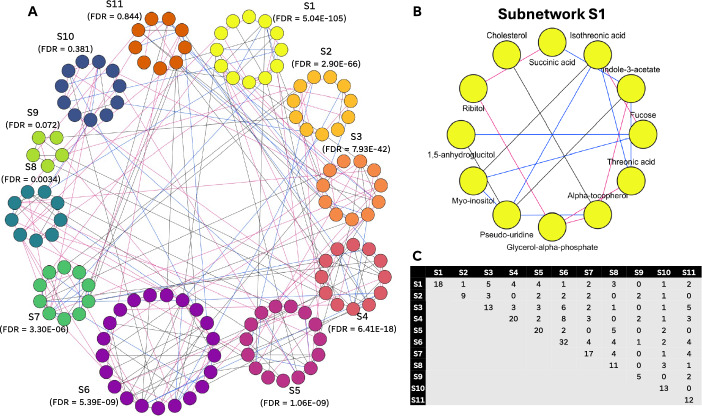
Visualization and interpretation of the T1D metabolic modules. DNEA analysis result for a subset of TEDDY metabolomics data. A. Clustered partial correlation network showing the enrichment analysis results. Blue, pink, and black lines represent edges present in either controls, cases, or both, respectively. Eight out of eleven shown subnetworks were found to be significant (FDR < 0.05). B. Detailed view of the top significant network (S1). C. Number of inter- and intra-subnetwork edges across eleven subnetworks

*OUTPUT—*clusterNet() returns the *DNEAobj* object after populating the consensus_clustering slot with the results of clusterNet(). Subnetwork membership for each node is also appended to the node list stored in the node_list slot.

Step 4. Enrichment analysis and network visualization

#### Enrichment analysis via NetGSA

The subnetworks obtained through consensus clustering can be tested for enrichment across the experimental conditions using the NetGSA algorithm [19].

*INPUT—*the runNetGSA() function takes as input a *DNEAobj* object created in *STEP 3.2*. NetGSA takes into account differential expression of individual metabolites, the strength of the network edges, and the network structure to determine enrichment [19]. Identification of metabolic modules within the networks via consensus clustering (*STEP 3.2*) is required if the user wants to perform enrichment analysis using NetGSA.

This approach to pathway analysis utilizes all the available data and supports the identification of potentially novel interactions between identified, un-identified, exogenous, and secondary metabolites. It also facilitates enrichment analysis for the constructed metabolic modules across two experimental conditions.

*OUTPUT—*This step populates the netGSA_results slot of the *DNEAobj* object with a *NetGSAresults* object containing information about each subnetwork, including number of edges/nodes and statistical significance of the enrichment result for each module.

#### Network visualization

Visualizing the constructed networks is an important step in biological interpretation. Several visualization methods are available to the user. The wrapper function, plotNetworks(), utilizes the igraph R package to visualize the experimental group networks or each of the subnetworks. The structure of the resulting output from DNEA also facilitates easy visualization of the biological networks using the igraph R package or commonly used external tools (e.g., Cytoscape [24], Metscape [15]). The function getNetworkFiles() takes as input the *DNEAobj* object and saves two files formatted for visualization in Cytoscape and Metscape: an edge list containing the metabolite pairs and partial correlation values, and a node list containing the differential status of each metabolite along with its statistical significance and subnetwork membership. Finally, an igraph adjacency graph object created during clustering is available in the consensus_clustering slot of the *DNEAobj* object and accessed with the adjacencyGraph() function for easy plotting with the igraph R package.

## Results

To illustrate the use of the DNEA R package, we used publicly available metabolomics data from the TEDDY study [25]. TEDDY Is a prospective case–control study that sought to understand environmental causes of T1D. High-risk infants, as defined by the presence of HLA-DR and HLA-DQ genotypes, were followed every 6 months and samples were collected until development of T1D or their 15th birthday, whichever occurred first. Here we considered two endpoints evaluated by the study: the presence of islet auto-antibody (IA), and the development of T1D. The Metabolomics data obtained from the Metabolomics Workbench [26] contained 11,560 samples from 1818 subjects, 3525 samples in the T1D arm, and 9024 samples in the IA arm of the data set. 414 of the 1818 subjects tested positive for Islet auto-antibodies, and 50 subjects developed T1D.

To evaluate the effectiveness of the regularization steps implemented in the DNEA package, unnormalized peak intensity values adjusted for age and sex were used as input. DNEA analysis was performed on IA and T1D datasets using all samples, followed by random sampling without replacement to obtain datasets containing 50%, 10%, and 5% of all samples. For each subset of the data, we evaluated the total number of edges, the number of shared edges, and the number of edges per condition. The results of these analyses are presented in Table 1. As the number of samples in the dataset decreased, both the total number of edges as well as the number of edges specific to each condition decreased, resulting in a sparser network.

**Table 1:** T1D and IA analysis results

Dataset	% of total samples	# SAMPLES			# EDGES			
		TOTAL	Control	Case	TOTAL	Shared	Control	Case
T1D	100%	3525	2688	837	3400	1722	969	709
	50%	1763	1344	419	2453	1150	613	690
	10%	353	269	84	1650	471	573	606
	5%	177	135	42	1483	376	551	556
IA	100%	9024	6984	2040	4579	2812	993	774
	50%	4512	3492	1020	3124	1723	712	689
	10%	903	699	204	1981	745	607	629
	5%	452	350	102	1555	474	485	596

The number of samples and edges per experimental group in the T1D and IA arms of the TEDDY dataset are shown. The λ parameter was optimized using the BIC tuning protocol and stability selection was performed without additional subsampling

Next, we repeated the analysis employing the subsampling protocol for the larger group (controls) during stability selection (Step 2.2) to determine its behavior with decreasing sample size. Similar to the experiments performed without subsampling, reducing the number of samples in a dataset resulted in sparser networks compared to the larger counterpart (Table 2).

**Table 2:** T1D and IA analysis results with subsampling of a larger group

Dataset	% of total samples	# SAMPLES			# EDGES			
		TOTAL	Control	Case	TOTAL	Shared	Control	Case
T1D	100%	3525	2688	837	3040	1596	1011	433
	50%	1763	1344	419	2086	1073	607	406
	10%	353	269	84	1523	524	519	480
	5%	177	135	42	1489	437	479	573
IA	100%	9024	6984	2040	4174	2629	1109	436
	50%	4512	3492	1020	2764	1620	751	393
	10%	903	699	204	1811	742	609	460
	5%	452	350	102	1398	491	473	434

In both cases, the λ parameter was optimized using the BIC tuning protocol during network construction with the getNetworks() function, and subsampling of the larger group was performed during stability selection. The sample distribution across the experimental groups, the total number of edges, the shared edges between networks, and the edges specific to each experimental condition in the T1D and the IA data are shown

Subsampling had a much greater effect on the case networks, reducing the.

case-specific and shared edges significantly more than the control-specific edges in both datasets. The stark difference in sample sizes across groups resulted in over-representation of the control samples in each random sample during stability selection and an overestimation of the selection probability of certain edges in the case network, leading to erroneous edge selection. Balancing the groups removed unstable or false-positive edges previously identified in the case network and resulted in more accurate and interpretable networks. 

Next, we ran the DNEA R on a subset of the TEDDY data that included measurements for 134 metabolites across 322 samples and visualized the resulting networks. The samples included 50 T1D cases obtained at the final visit when the participants were diagnosed with the disease and 272 sex- and age- matched controls obtained at a visit closest to the matched cases’ final visit. Due to the small sample size and uneven sample distribution across the experimental condition, subsampling was utilized during stability selection and the λ parameter was approximated during network construction. Approximating λ led to increased regularization and consequently resulted in sparser networks (Fig. 1). 

Network visualization was performed using the Cytoscape program (v3.10.0) by importing the edge list generated using the getNetworkFiles() function in the DNEA R package. The resulting network contained 290 edges. Of these, 112 edges were specific to T1D cases, 70 edges were specific to the control group, and 108 edges were shared between the two conditions (Fig. 2A). See Supplementary Table 1 for the complete DNEA results. Network clustering resulted in thirteen subnetworks that contained 132 of the 134 metabolites. Eleven subnetworks that contained 5 or more metabolites were tested for enrichment. Eight of these (S1–S8) were found to be statistically significant (FDR < 0.05). The significant subnetworks contained a number of metabolites that have been previously implicated in T1D. For example, S1 contained 5-annhydroglucitol, a known marker of glycemic control, threonic acid that is a hallmark of oxidative stress [27] and glycerol-alpha-phosphate that was found to be an important early predictor of T1D diabetes in TEDDY participants [28] (Fig. 2B). Other significant subnetworks contained additional markers of metabolic stress, e.g. methionine (S2) and cysteine (S7). The reduced levels of these metabolites and threonic acid are indicative of increased oxidative stress. Not all metabolites found in significant subnetworks have such direct connection to T1D. For example, indole-3-acetate (S1) is a gut-derived metabolite of tryptophan may be associated with the severity of hyperlipidemia, however it’s exact role in T1D has yet to be explored. Webb-Robertson et al. [28] identified eleven metabolites that were predictive of T1D in TEDDY study participants ages 3–9 months. Notably, ten of these are present in significant subnetworks identified by DNEA. The partial correlation networks generated by the package can provide context for building mechanistic hypotheses to explain the underlying physiological and pathological processes. Further, DNEA can be used to narrow down the list of metabolites that can be passed to predictive models, e.g. for biomarker selection. 

Upon inspection of the intra- and inter-subnetwork edges, we observed a higher proportion of intra-subnetwork edges (Fig. 2C), suggesting that the metabolites within a subnetwork are more correlated with each other than with metabolites in other subnetworks.

## Discussion

Rapid emergence of metabolomics as a high throughout science created a number of methodological challenges for data analysis, from processing raw MS data and removing degenerate features, mid- and high-level statistical, and bioinformatics analysis [29]. Some tools and approaches developed for the analysis of RNA seq and other omics data [30, 31] have been adopted for metabolomics. Publicly available tools developed for pathway analysis of RNA-seq data often rely on a variation of the gene set enrichment analysis algorithm (GSEA) [29]. This method was adopted for metabolomics data in Bioconductor R packages FELLA, metapone and Metabox [32–34], and web-based tools Metaboanalyst and MBrole [35, 36]. However, this approach translates poorly for several reasons. Metabolomics data often contain exogenous metabolites and unknown compounds that cannot be mapped to metabolic pathways. Further, pathway databases contain limited information about lipid and secondary metabolic pathways. As a result, a lot of experimentally measured compounds cannot be mapped to pathway(s) and must be excluded from the analysis. This limits the usability of traditional pathway-based enrichment analysis methods for metabolomics data. The DNEA R package described here can help overcome this limitation. It uses partial correlations of normalized metabolite intensity values to build data driven networks, followed by consensus clustering to define highly correlated metabolic modules within that network. The resulting metabolic modules are tested for enrichment across the experimental groups using the built-in algorithm. This approach circumvents the requirement for well annotated metabolic databases for pathway analysis, and expands the ability to analyze exogenous-, secondary-, and unknown- metabolites. By using data-driven modules instead of predefined pathways, DNEA utilizes both known and unknown metabolites in the analysis. This approach enables the discovery of potentially previously unknown reaction partners and other important biological interactions. It may aid in metabolite identification and accelerate the study of discovered metabolites. Several existing computational tools utilizing pairwise mass differences, retention times and pairwise Pearson/Spearman correlations of feature intensities can be employed downstream of DNEA to help evaluate metabolites at the biochemical reaction level [37, 38]. For well annotated datasets, the user may provide pathway definitions from their preferred database and test these for enrichment. In both cases, NetGSA accounts for the differential correlation of metabolites in a given pathway to improve the accuracy of enrichment results.

### DNEA enhancements

The implementation of the DNEA R package described in this study has several enhancements compared to Filigree [16]. The modular design of the DNEA R package allows users to customize the workflow and adopt it to their analysis needs. For example, the user may choose to normalize the data using their preferred methods, bypassing the auto-scaling step.

**Fig. 3:**
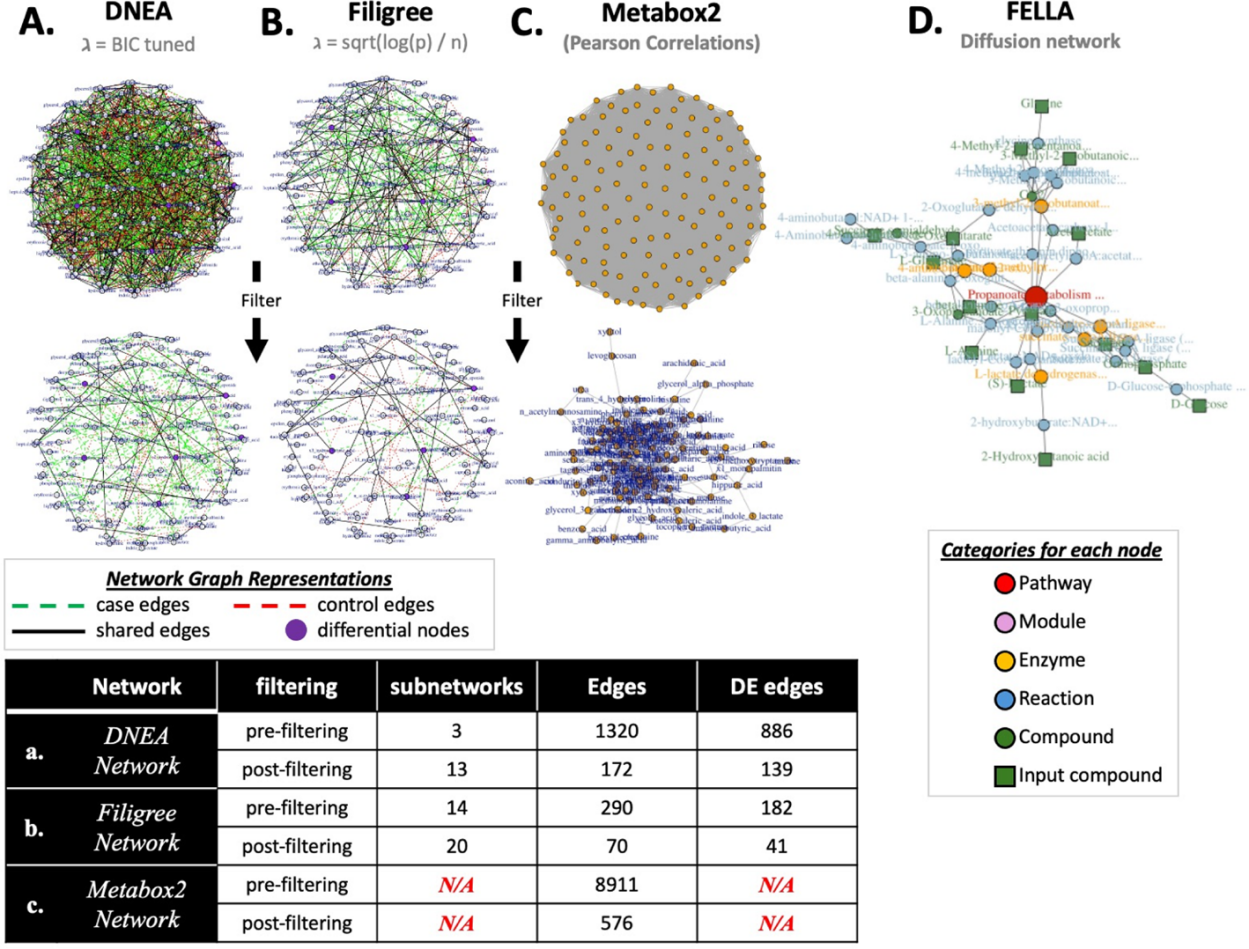
Comparison of the DNEA R package with Filigree, Metabox2 and Fella. The networks were constructed using a subset of IA data from the last visit for each patient. The DNEA, Filigree and Metabox2 networks were filtered to include edges with an absolute value of correlation coefficient greater than or equal to 0.2. A. DNEA network. B. Filigree network. C. The Metabox2 network. The igraph R package was used to plot the network. D. The FELLA R package was used to create a network using KEGG pathway definitions and a list of named metabolites from IA data

The user now has more flexibility in optimizing the regularization parameter, λ. The ability to control the λ parameter in network construction improves the sensitivity of analyses for both small and large datasets. To illustrate the improvements in the DNEA R package relative to Filigree, we constructed networks for the last visit for each patient from the IA data set. First, the network was created utilizing the advancements implemented in the DNEA R package (i.e., subsampling was increased to 1000 replicates and the λ parameter was turned for each group using the BIC tuning protocol during joint estimation) (Fig. 3A). Next, we used to the same data to create the network utilizing default Filigree parameters (i.e., the subsampling protocol used 500 reps of stability selection, and the λ parameter was estimated using $\uplambda = \sqrt{\frac{\text{log}\left(m\right)}{n}})$(Fig. 3B). Tuning λ finds the theoretically optimal value and generally results in less model regularization as well as more sensitivity to detect edges compared to estimating the λ value. Tuning resulted in 172 edges detected with an absolute partial correlation value greater than or equal to 0.2 versus the 70 identified by estimating λ. 139 of the 172 edges were differential when tuning versus 41 of 70 when λ was estimated. The increase in sensitivity gained by tuning the parameter is most noticeable in the experimental condition with the smaller sample size, in this case the IA positive group, as seen by the increase in green edges after filtering (Fig. 3A, B). Utilizing the BIC protocol for network construction increases the sensitivity of DNEA, however, a large dataset is required (~ 500 or more samples per group). A user may also opt to utilize their preferred external method for hyperparameter optimization and then specify the λ value to be used in analysis. For smaller datasets, an approximation of the λ parameter following Eq. 3 should be used, as it reduces false positive identification of unstable edges.

Further, in settings with grossly imbalanced sample sizes across groups, subsampling each group during stability selection reduces bias toward the larger group. In practice, this additional step increases the specificity of the networks, making them more sparse. In previous experiments using the metabolomics data from the Framingham heart study, subsampling of the larger group reduced the total number of edges across both conditions, but the most marked decrease occurred in the larger group [16]. This result follows the expectation that the selection probabilities calculated in stability selection more equally represent both groups, so the network corresponding to the larger group should become more sparse. During the analysis of the TEDDY dataset described here, the total number of edges across the network decreased as expected, however, the largest reduction of network connections occurred in the smaller group, indicating the presence of weak, less stable edges.

Additional new features include the implementation of network filtering through the filterNetworks() function that aids interpretation by controlling the network density. Furthermore, filtering to remove weak edges prior to consensus clustering also improves performance of the algorithm by focusing the analysis on the most biologically relevant interactions and reducing noise in the dataset. To that end, the user can now adjust the specificity of subnetwork identification during consensus clustering. Network filtering along with the ability to adjust the stringency of consensus clustering through the tau parameter highlight metabolite-metabolite interactions with the strongest correlations and provide more control over subnetwork identification. Increasing the value of tau reduces the possibility of weak metabolite associations being included into a given subnetwork, thereby improving the power of enrichment analysis by limiting tested modules to relevant metabolite-metabolite interactions only. The number of identified subnetworks tends to increase as tau increases, while the average size of each subnetwork decreases. The consensus clustering algorithm, in conjunction with enrichment analysis, enable the identification of important metabolic modules that are differentially enriched across the experimental condition.

An improved feature aggregation algorithm has also been included in this implementation. In the correlation-based and hybrid methods, the user now specifies a Pearson correlation threshold for aggregation. Feature aggregation (*STEP 1.2*) is extremely useful in reducing complexity of the analysis, resulting in more interpretable networks. L1 regularization often keeps one compound from a highly correlated group of metabolites in the dataset and the remainder are removed from the model. Many studies in metabolomics/lipidomics often contain highly correlated compounds of a specific class, such as fatty acids, that may be affected. In this case, aggregating those compounds into a single feature allows the user to keep information from all of these compounds in the model and provides more information about connectivity of the networks than would otherwise be available.

### Comparison of DNEA with other tools

To further illustrate the features of the DNEA R package we compared it to several available open-source tools. These include the Fgsea (fast implementation of GSEA algorithm), FELLA, Metapone, Metabox2, Metaboanalyst, MBrole [32, 33, 35, 36, 39, 40], and our own Java-based tools, CorrelationCalculator and Filigree [15, 16, 21] (Table 3).

**Table 3:** Comparison of the DNEA R package with existing bioinformatics tools for interpretation of metabolomics data

	Input	Number of experimental conditions	Adduct/fragment removal	PA considers unknown metabolites?	Constructing data-driven networks	PA using predefined pathways	PA using data driven metabolic modules	HPC compatible
DNEA R package	*m x n* matrix of peak intensity/concentrations	2	*through external tools*	✓	Graphical LASSO (partial correlations)	✓	✓	✓
Fgsea	*Ranked list of differential features*	2	*through external tools*	❌	❌	**✓**	❌	❌
FELLA	*m x n* matrix of peak intensity/concentrations	2	*through external tools*	❌	Diffusion Algorithm	**✓**	❌	**✓**
Metabox2	*m x n* matrix of peak intensity/concentrations	2	*through external tools*	❌	Pathway-defined/ Weighted correlations	**✓**	❌	❌
Metapone	*m x n* matrix of peak intensity/concentrations	2	**✓**	❌	❌	**✓**	❌	**✓**
Metabo-Analyst	*m x n* matrix of peak intensity/concentrations	2	*through external tools*	❌	❌	**✓**	❌	*N/A*
MBrole	*List of input features*	2	*through external tools*	❌	❌	**✓**	❌	*N/A*
Correlation calculator	*m x n* matrix of peak intensity/concentrations	1	*through external tools*	**✓**	De-sparsified graphical LASSO	❌	❌	❌
Filigree	*m x n* matrix of peak intensity/concentrations	2	*through external tools*	**✓**	Graphical LASSO (partial correlations)	❌	**✓**	❌

The DNEA R package utilizes the GLASSO algorithm and a consensus clustering approach to construct metabolic modules that can be tested for enrichment across two experimental conditions. DNEA is the only package available that constructs and subsequently enables enrichment analysis across data-driven metabolic modules. This method utilizes partial correlations, estimated by the GLASSO algorithm, to remove confounding associations so that direct reaction partners can be identified. Using NetGSA for enrichment analysis on these metabolic modules allows the entire dataset to be utilized, including secondary, exogenous, and unknown metabolites that do not map to pathway databases

In addition to feature-by-feature comparison presented in Table 3 we compared the networks generated by DNEA R package, Metabox2 and Fella (Fig. 3A, C, D). Metabox2 is a suite of computational tools for the analysis of metabolomics data. It includes the functionality for correlation-based network analyses and pathway enrichment. A user can calculate Pearson or Spearman correlations for every edge and export the data for network graphing to their preferred software. While marginal correlations have been previously used to construct and analyze biological networks, they tend to be very dense due to the inclusion of edges representing both direct and indirect correlations. The latter can confound the results making identification of biologically relevant interactions more difficult. In contrast, DNEA calculates partial correlations, controlling for potential confounding effects from other metabolites in the dataset. Further, DNEA can estimate edges that are differential between two experimental conditions. We performed Metabox2 correlation analysis using the same dataset that was used in DNEA analysis. The Metabox2 network was filtered to include edges with an absolute value of Pearson correlation coefficient greater than or equal to 0.2. Metabox2 network (Fig. 3C) was considerably more dense (576 edges vs, 172 edges in the DNEA network) making the interpretation more difficult. In contrast to DNEA, MetaBox2 does not use clustering to identify subnetworks. Instead, it can perform pathway analysis using pre-defined pathways tying biological interpretation to well annotated metabolites.

Another open-source network analysis tool for metabolomics data, FELLA, builds a hierarchical representation by integrating pathways, enzymes, genes, and metabolites from the Kyoto Encyclopedia of Genes and Genomes (KEGG) database. This package takes as input a list of KEGG IDs for the metabolites present in the data. In contrast to DNEA, FELLA uses information from the KEGG database to build the network (Fig. 3D). Thus, FELLA requires KEGG IDs, limiting the analysis to the metabolites that are annotated in KEGG. As a result, only 19 out of 174 metabolites present in the TEDDY data were included in the analysis.

## Conclusions

The ever-growing complexity of metabolomics studies requires more and more computing resources to analyze the collected data. The implementation of the DNEA R package presented here allows for the analysis of large datasets containing unannotated or poorly annotated metabolites in a data-driven manner. DNEA R supports the use of cloud services or a high-performance computer running the R programming language. A framework for parallel processing has been built into the most computationally expensive steps—model tuning and stability selection—which reduces the time burden of the analysis while keeping the memory footprint at a minimum. Altogether, the new features implemented in the DNEA R package allow for the analysis of datasets that, due to mathematic instability, unacceptable power due to more features than samples, or insufficient computing resources, were intractable. In addition, the modulable nature of the package enables the user to apply the most appropriate workflow to their data and provides more control over the analysis than ever before.

## Materials and methods

**Software availability:** The DNEA R package is available to download on GitHub (http://www.github.com/Karnovsky-Lab/DNEA/).

### Metabolomics data

The metabolomics data used in our analyses were obtained from the Metabolomics Workbench (ST001386 and ST001636). These data are also included in the DNEA R package. The data were filtered to exclude metabolites that were missing in more than 30% of the samples. For the remaining metabolites, all missing values were imputed with the median value of each metabolite. Samples from individuals that started as an IA or T1D control and later became an IA or T1D case were not included into the analysis. Each dataset was adjusted for age and gender by taking the residuals of a linear regression model and shifting the values so that the minimum is zero for each metabolite, respectively.

### Data analysis and visualization

Each DNEA experiment was performed on the Great Lakes cluster at the University of Michigan using 120 cores over 6 nodes. Data visualization was performed using Cytoscape (v3.10.0) by importing the node and edge lists generated by the DNEA function, getNetworkFiles().

## Supplementary Information

Supplementary material 1.

Supplementary material 2.

## Data Availability

The DNEA R package described in the manuscript is available to download on GitHub (http://www.github.com/Karnovsky-Lab/DNEA/). The datasets used to test DNEA R package were obtained from the Metabolomics Workbench (https://metabolomicsworkbench.org). Accession numbers ST001386 and ST001636.

## Abbreviations

DNEA: Differential Network Expression Analysis
TEDDY: The Environmental Determinants of Diabetes in the Young
PCN: Partial Correlation Network
JEM: Joint Estimation Method
m: Number of features in a dataset
n: Number of samples in a dataset
BIC: Bayesian Information Criterion
Glasso: Graphical Lasso Algorithm
λ: Hyperparameter: Lambda
Ρ: Hyperparameter: Rho
GSEA: Gene set enrichment analysis
HPC: High-performance cluster
IA: Islet Auto-Antibody
T1D: Type I Diabetes
