# Supplementary material for: DNEA: an R package for fast and versatile data-driven network analysis of metabolomics data
Source: BMC Bioinformatics. 2024 Dec 18;25:383. doi: 10.1186/s12859-024-05994-1 (PMC11657348; doi:10.1186/s12859-024-05994-1)
Supplement: Supplementary file 1 — Supplementary material 1. [file 12859_2024_5994_MOESM1_ESM.pdf]

Supplemental Figure 1.

Hyperparameter Tuning

a.

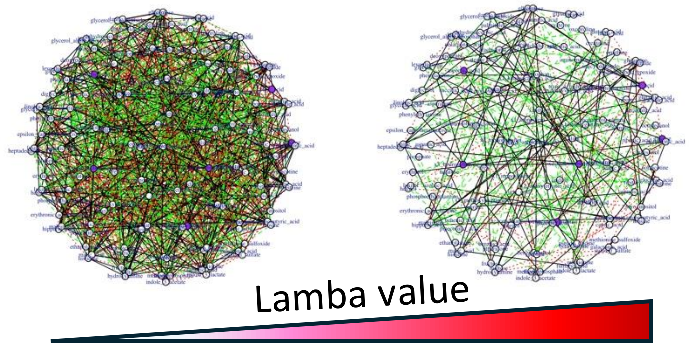

As the Lambda value increases, regularization of the model increases, thereby removing the most unstable edges from the network

b.

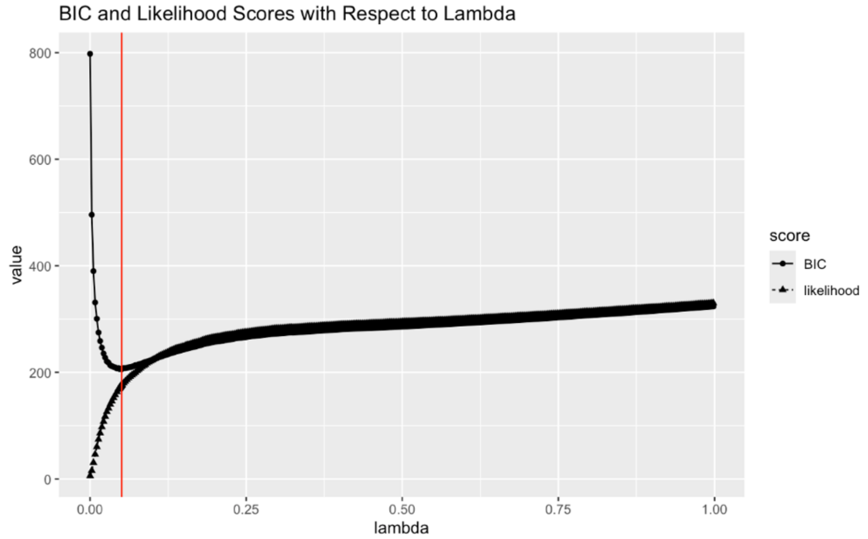

c.

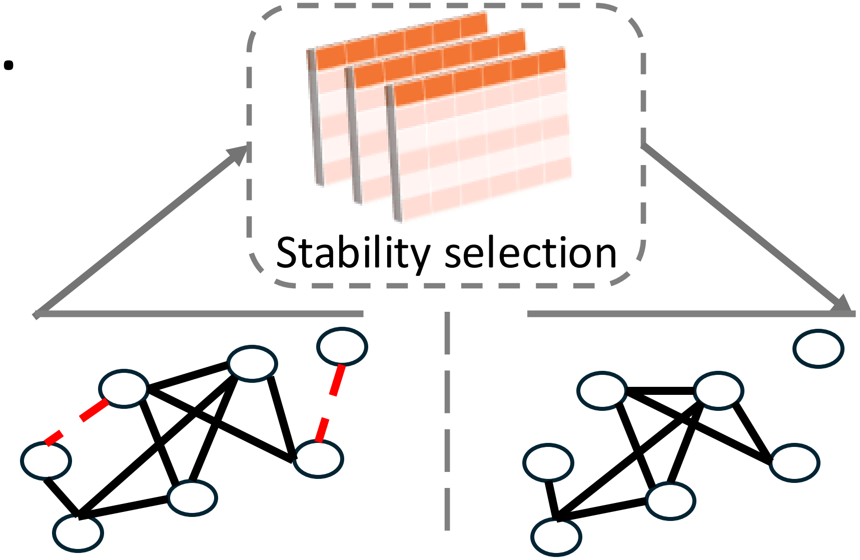

Supplemental Figure 1. Visual Representation of regularization in DNEA. (A) Regularization, and as a result network density, are heavily influenced by the ƛ parameter. ƛ can take on any value between 0-1. As the value increases, regularization increases and network density decreases. **(B)** DNEA utilizes Bayesian Information-Criterion (BIC) scores to select the optimal model. Many ƛ values are tested for model fitting, and the value that results in the minimum BIC score is selected as the optimal model. **(C)** Stability selection is performed by randomly sampling the input data many times and calculating the probability that an edge is identified in a random sample of data. These probabilities are then used to weight the model. This method improves model accuracy by removing unstable edges from the networks

Supplemental Figure 2.

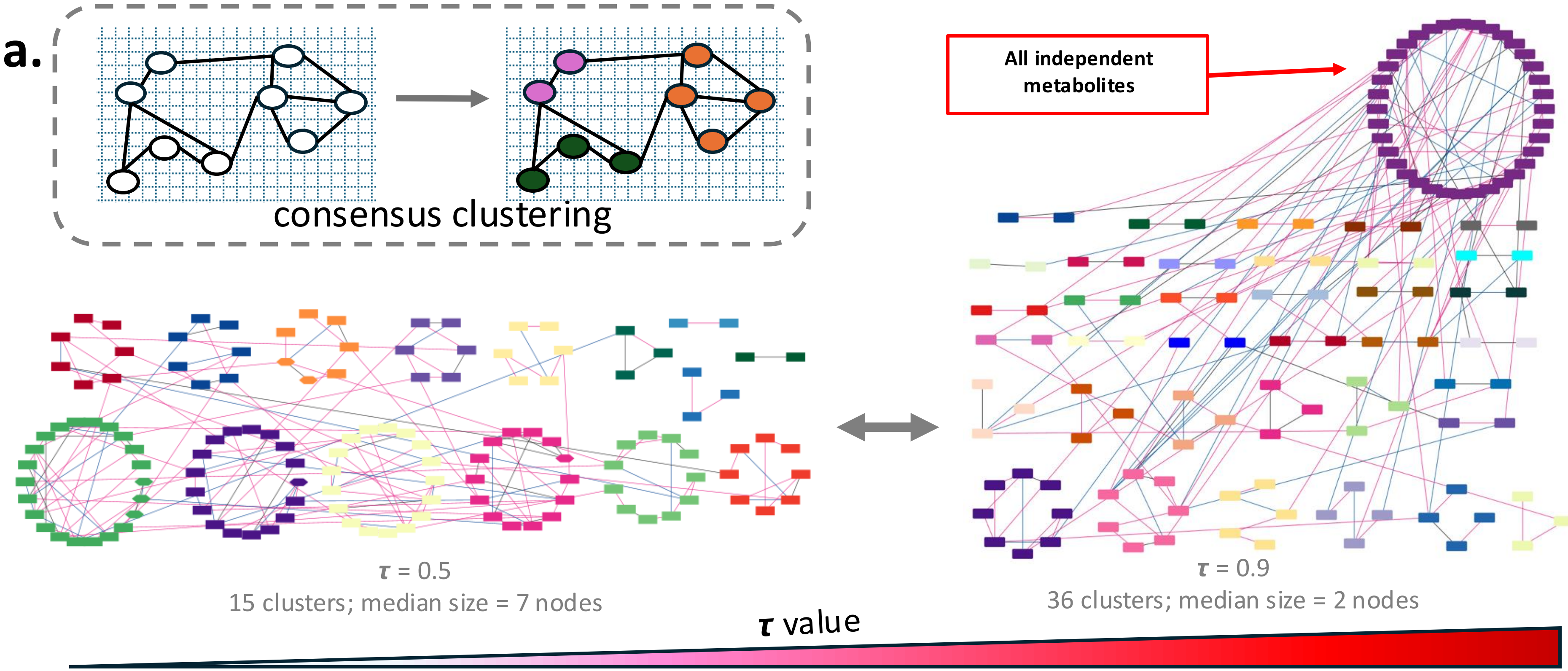

Supplemental Figure 2. Visual representation of consensus clustering. Consensus clustering utilizes 7 algorithms from the igraph R package: cluster_edge_betweeness, cluster_fast_greedy, cluster_infomap, cluster_label_prop, cluster_louvain, cluster_walktrap, and cluster_leading_eigen, to construct metabolic modules of highly correlated metabolites within the broader networks. The stringency of node inclusion into a module is controlled by the consensus threshold, tau (τ), which can take on a value between 0.5-1. As the value of τ increases, the agreement requirement for a given nodes inclusion into a subnetwork is more stringent. In practice as the value increase, the number of identified metabolic modules increases and the number of nodes per module decreases.
